# Transjugular Intrahepatic Portosystemic Shunt for Budd–Chiari Syndrome: A Single-Centre Experience

**DOI:** 10.3390/jcm13195858

**Published:** 2024-10-01

**Authors:** Faisal Joueidi, Amnah Alhanaee, Hamad Alsuhaibani, Ali Albenmousa, Ahmad Joueidi, Ahmed Elhassan, Abdallah Nabeel Nasir, Kris Ann Hervera Marquez, Saad Alghamdi, Waleed Al Hamoudi, Saad Abualganam, Dieter Broering, Khalid Ibrahim Bzeizi

**Affiliations:** 1 College of Medicine, Alfaisal University, Riyadh 11451, Saudi Arabia; ahmadjoueidi123@gmail.com (A.J.); ahmed2000alhassan@gmail.com (A.E.); abdullah.nasr3@gmail.com (A.N.N.); 2 Department of Liver & Small Bowel Health Centre, Organ Transplant Centre of Excellence, King Faisal Specialist Hospital & Research Centre, Riyadh 11451, Saudi Arabia; amnahalhanaee@gmail.com (A.A.); albenmousa@kfshrc.edu (A.A.); krhervera@kfshrc.edu.sa (K.A.H.M.); mdisaad@kfshrc.edu.sa (S.A.); awalhamoudi@kfshrc.edu.sa (W.A.H.); dbroering@kfshrc.edu.sa (D.B.); kbzeizi@kfshrc.edu.sa (K.I.B.); 3 Radiology Department, King Faisal Specialist Hospital & Research Centre, Riyadh 11451, Saudi Arabia; halsuhaibani@kfshrc.edu.sa (H.A.); sabualganam@kfshrc.edu.sa (S.A.)

**Keywords:** Budd–Chiari syndrome, transjugular intrahepatic portosystemic shunt, TIPS revision, covered stent, liver transplantation (LTx)

## Abstract

**Background**: Despite several challenges in clinical management, there has been significant progress in understanding the aetiology, natural history and outcomes of Budd–Chiari syndrome (BCS) treatments. This study aims to evaluate the outcomes of transjugular intrahepatic portosystemic shunt (TIPS) using covered stent in management of BCS. **Methods**: We conducted a retrospective analysis of 70 BCS patients who underwent TIPS using covered stents between January 2010 and December 2022 at a single tertiary liver transplant centre. Patients’ clinical features, laboratory parameters, and imagine findings were collected before and after TIPS. The primary endpoint was overall survival. **Results**: TIPS was performed on 70 patients with BCS out of a total of 88 patients. The remaining patients (18) underwent liver transplantation. The mean age was 37.7 ± 11.2 years at time of diagnosis and the majority were female (64.35). The most common symptoms and signs at presentation were abdominal pain, jaundice, ascites, and variceal bleeding. Over a median followup of 76 months, the survival rates at 1, 3, and 5 years were 98.8%, 97.9%, and 97.7%, respectively. Patients who underwent TIPS alone had better survival that patients with BCS who required liver transplantation (LTx) (*p* = 0.003). **Conclusions**: In our study TIPS provided a highly effective treatment option for BCS patients. The long-term favourability of the outcome was not impacted by the need for repeated TIPS revision. Use of covered stents was instrumental in reducing shunt dysfunction rates. Prospective and larger studies are needed to further optimize therapeutic strategies in this challenging population.

## 1. Introduction

Budd–Chiari syndrome (BCS) is a rare clinical condition first described by George Budd, a British physician, in 1845 and later described by Austrian pathologist Hans Chiari in 1898. Clinical manifestations occur as a result of obstruction of the hepatic venous outflow, which may include small hepatic venules, larger hepatic veins, or the inferior vein cava (IVC), which results in the development of liver congestion, hepatomegaly, ascites, and acute liver failure in severe cases [1,2]. Primary BCS occurs because of venous manifestations, including thrombosis, web-like formations, or inflammation. This type is often associated with more immediate and profound symptoms. Extra-luminal compression triggers secondary BCS because of several factors, including tumours, abscesses, cysts, or pericardial conditions [3,4]. The aetiology is associated with pro-thrombotic states, including myeloproliferative neoplasms, congenital coagulation disorders, and malignancies. The manifestation of BCS varies depending on the degree and severity of hepatic venous outflow obstruction and existing collateral veins to relieve pressure on the liver’s vessels. BCS is categorized into the following four types: fulminant, acute, subacute, and chronic [5]. The rarer fulminant type may lead to rapid-onset hepatic encephalopathy after jaundice appears. Patients with acute BCS experience severe ascites and liver damage [5]. The most frequent subacute BCS may cause few to no ascites if compensated by collateral veins. Chronic BCS manifests symptoms of portal hypertension, such as ascites and variceal bleeding [5,6]. If left untreated, the outflow obstruction of the disease has an unfavourable natural course, with a mortality rate of 50% in 2 years and a survival rate of <10% in untreated patients within 3 years. Venous outflow obstruction leads to hepatic congestion and fulminant fibrosis, which develops within 3 months. TIPS is an interventional radiology procedure that has affected the management of BCS. It was first described by Josef Rösch in 1969 [7], and Rössle et al. reported the first successful TIPS [7,8]. It involves creating a low-resistance channel between the hepatic vein and the intrahepatic portion of the portal vein, therefore relieving the obstruction and reducing portal pressure. TIPS has revolutionized the approach towards the management of refractory ascites, variceal bleeding, and hepatic venous outflow obstruction, offering a less invasive alternative to surgical shunting procedures [9]. In BCS, TIPS plays a critical role in alleviating symptoms, improving liver function, and enhancing overall survival rates. Studies have shown that TIPS not only provides symptomatic relief but also addresses the pathophysiological aspects of the syndrome by restoring hepatic venous flow, which can halt disease progression and reduce the necessity of LTx in selected cases [8,10]. TIPS is associated with additional challenges for patients with BCS. The lack of normal hepatic veins and caudate lobe hypertrophy can pose technical challenges. TIPS dysfunction is more common, given the increased risk of thrombosis related to the underlying aetiologies of BCS. Lifelong anticoagulation and the use of covered stents have improved patency rates and reduced the risk of shunt dysfunction, further establishing TIPS as a viable and effective therapeutic option for BCS patients [11]. The data on TIPS management of BCS in our region are limited, and this study aims to report the short and long-term outcomes of TIPS intervention observed in our patients with BCS.

## 2. Material and Methods

This is a descriptive retrospective cohort study that included adult patients with BCS who were managed with TIPS insertion only at King Faisal Specialist Hospital and Research Centre (KFSH&RC), Riyadh, Saudi Arabia, during the period from January 2010 to December 2022. Patient data were retrospectively collected from the Citrix system of KFSH&RC. Demographic data, signs and symptoms at presentation, risk factors, complications, and outcomes of treatment were recorded, together with laboratory data, radiological findings, and co-interventions. Diagnoses were made according to the European network for vascular disorders of the liver (En-Vie) criteria and the last Baveno consensus, which is based on an image that shows an obstructed venous tract [8]. Doppler ultrasonography, Magnetic Resonance Imaging (MRI), or Computerized Tomography (CT) were used for diagnosis. Whenever available, detailed haematological investigations for hypercoagulable state were reported, including testing for Factor V Leiden mutation, Janus Kinase 2 (JAK-2) mutation, prothrombin G20210A gene mutation, antiphospholipid antibody syndrome, paroxysmal nocturnal haemoglobinuria, C and S protein deficiencies, and Antithrombin III (AT III) deficiency. The severity of liver disease was assessed using the Model of End Stage Liver Disease-Na (MELD-Na) score. The institutional protocol for TIPS insertion was followed, and all procedures were performed by a single experienced interventional radiologist using a standard technique with a 10 mm diameter covered metal stent [11,12,13]. The portal vein was accessed directly from the IVC with the absence of sufficient hepatic vein (HV) stump or remnant. Anticoagulation therapy was started post TIPS according to a multi-disciplinary decision made by a hepatologist, haematologist, and radiologist. The primary measured outcome was overall survival after TIPS, while secondary outcomes were liver-related morbidity, the need for LTx, and the incidence of TIPS dysfunction (stenosis or occlusion). This study was performed in accordance with the Declaration of Helsinki and approved by the institutional ethical committee of King Faisal Specialist Hospital and Research Centre (reference number: #2121012/07/07/2024). Informed consent was waived by the ethical committee due to the retrospective nature of the study.

## 3. Statistical Analysis

Descriptive statistics are presented as the mean, standard deviation (SD), and range. Statistical significance was set at a *p* value of less than 0.05. A paired t-test was used for the analysis of laboratory data. Kaplan–Meier survival analysis was used to estimate the survival function from the data. Analyses were conducted using Microsoft Excel and SPSS (SPSS Inc., Chicago, IL, USA).

## 4. Results

A total of 88 patients with BCS were reviewed. Seventy patients were managed with TIPS insertion alone, while 18 patients were managed with liver transplantation and were used as a comparative group. Most of the patients in the TIPS group were females (64.35%), with a mean age of 37.7 ± 11.2 years. The baseline characteristics are summarized in Table 1.

BCS diagnosis was made based on a Doppler US and/or CT scan of the abdomen. All patients received anticoagulation treatment after diagnosis.

**Figure 1:**
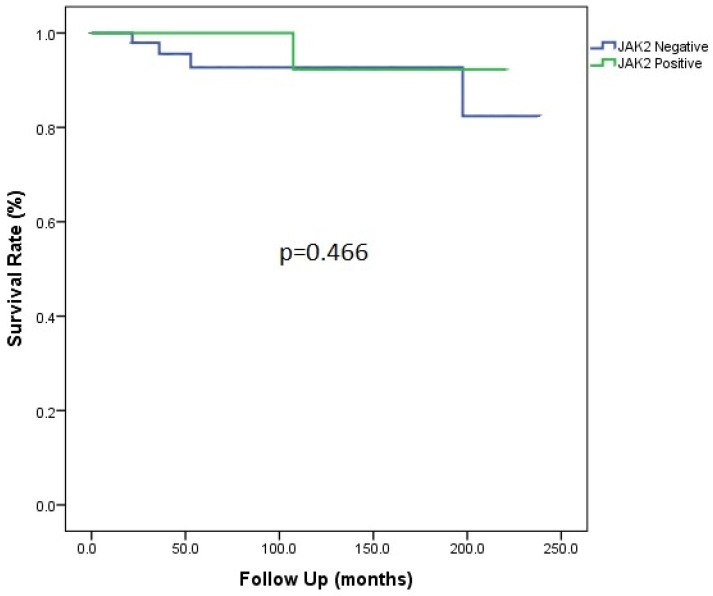
Patient survival according to JAK2 mutation status.

The most frequently reported symptoms and signs at the time of diagnosis were abdominal pain (88.6%), jaundice (88.6%), and ascites (71.5%) (Table 2). The aetiology of BCS was identified in 63% of the cases, and myeloproliferative disorders defined by positive JAK2 mutation were the most common identifiable cause. Other causes of hypercoagulable state in this cohort are summarized in Table 1. The presence of JAK2 mutation in BCS patients was not associated with increased mortality (*p* = 0.466) (Figure 1). The median MELD score at the time of diagnosis was 18 (6–36), mostly driven by high total bilirubin. The Child–Pugh score was also calculated, although we believe it does not accurately represent the severity of liver disease in these patients and the whole BCS cohort (88 cases); a total of 19 patients (21.6%) were classified as Child A, 50 patients (56.8%) as Child B, and 19 patients (21.6%) as Child C.

While TIPS was performed alone in 70 patients, 5 patients (5.7%) underwent TIPS followed by LTx due to TIPS failure, and 13 patients (14.8%) underwent LTx directly. Malfunction of TIPS (stenosis/occlusion) was reported in 22 patients (31.4%) in the TIPS-alone group, requiring one or more revisions. Revision was successful in all cases, as confirmed by adequate blood flow through the stent via doppler US.

Over a median followup of 76 months (0–239 months), the overall survival rates at 1, 3, and 5 years in the TIPS-alone group were 98.8%, 97.9%, and 97.7% respectively. No major complication was noted, except in one patient (1.4%), who developed hemoperitoneum and was managed conservatively.

**Figure 2:**
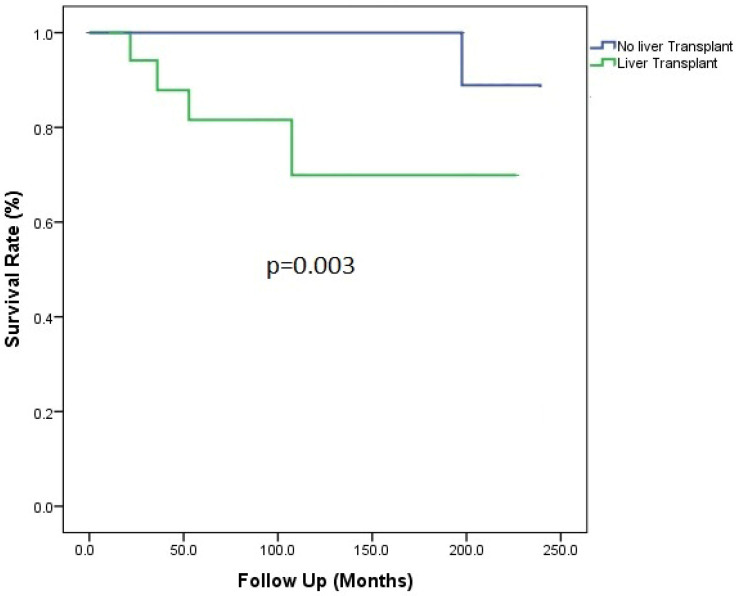
Patient survival according to liver transplant status.

Among patients who underwent LTx with or without prior TIPS, four patients (22.2%) died in the first year because of liver-related complications, including refractory haemorrhagic shock, bile leak, and multi-organ failure. Better survival was observed in the TIPS-alone group when compared to the LTx group (*p* = 0.003) (Figure 2).

## 5. Discussion

In this study, we evaluated the clinical course and long-term outcomes of all BCS patients who were referred to our tertiary care centre and underwent TIPS. Overall, our patients were young and mostly female. This is similar to reports from other parts of the world. In our study, the most common presentations were abdominal pain and jaundice, followed by ascites, variceal bleeding, and acute liver failure. Sonavane et al. reported that the most frequently observed symptoms were ascites (100%) and variceal bleeding (78.57%). In their study, abdominal pain was the main presentation in only 19% of the patients. Jaundice was frequent (52.4%) but was less frequent than in our cohort [12]. Seijo et al. analysed the long-term outcomes of BCS under stepwise management [13]. Sixty-two patients underwent TIPS (39.5%) for refractory ascites (69%), liver failure (13%), and variceal bleeding (7%) [13]. Pagan et al. showed that the most common indications of TIPS were refractory ascites (59%), liver failure (22%), and upper GI bleeding (9%) [14]. In our study, the most common aetiology was myeloproliferative disorders, followed by liver cirrhosis, portal venous thrombosis, and antiphospholipid syndrome. Murad et al. from the Netherlands and Sonavane et al. from India also reported high rates of myeloproliferative disorders in their cohorts (39% and 40.4%, respectively) [12,15]. Pavri et al. from the United States reported that liver cirrhosis was more common in their cohorts than in ours (55.3%) [16]. Pagan et al. from Spain similarly reported high rates of antiphospholipid syndrome in their cohort (12.1%) [17].

Given the rarity of BCS, most published papers evaluating the outcomes of TIPS in BCS patients are small case reports and case series [18]. In Europe, TIPS is a widely used intervention for BCS, known for its high technical success and low complication rates [11]. Rössle et al. reported initial technical success in 33 out of 35 patients, with subsequent 1- and 5-year transplant-free survival rates of 93% and 74%, respectively [19]. Pagan et al. showed that BCS patients who did not improve with anticoagulation and underwent TIPS had 1-year and 10-year transplant-free survival rates of 88% and 69%, respectively [17].

The survival rates in our research were more favourable than those seen in a previous multi-centre European retrospective study involving 124 BCS patients treated with TIPS [20]. Other studies reported 5-year transplant-free survival rates of 77% to 100% following TIPS [13,21]. Despite the technical challenges in our cohort, the success rate of TIPS was observed to be 100%, without any procedurally related deaths documented. Xingshun et al. reported that 12 patients died following TIPS and TIPS revision because of massive variceal bleeding, anticoagulant-related intracranial haemorrhage, progressive liver failure, and duodenal perforation [18]. Sonavane et al. reported that a total of 11 patients died following the TIPS procedure because of haematological disorders, gastrointestinal bleeding, hepatic encephalopathy, and procedurally related mortality [12]. Qi et al. reported that the use of TIPS in BCS was associated with a mortality rate of 0–26%. The natural course of the disease is extremely unfavourable in untreated cases, with a mortality rate of 50% in 2 years and survival rate of 3 years [22].

Major procedurally related complications of TIPS are common and noticeable in most BCS patients. Xingshun et al. showed that three patients had developed intraperitoneal bleeding following the TIPS procedure [18]. Qi et al. estimated complication rates following the TIPS procedure to range from 0 to 56%, including bleeding, venous injury, capsule perforation, and kidney failure. Hayek et al. reported that almost 32% of BCS patients experienced early complications and 26% had developed peri-procedural complications [18]. Sonavane et al. showed that out of 42 patients, 8 developed recurrent ascites and 3 patients had hepatic encephalopathy following TIPS [12]. Tripathi et al. reported their experience with a total of 56 patients who underwent TIPS; almost 15% developed TIPS-associated encephalopathy [14]. In our study, only one patient experienced hemi-peritoneal bleeding, and all other patients were successfully treated without any further sequelae or severe post-operative complications.

TIPS dysfunction is a notable complication in BCS-TIPS patients because of their prothrombotic states secondary to thrombosis or pseudo-intimal hyperplasia, often leading to the recurrence of symptoms. To restore TIPS patency, multiple sessions of angioplasty and stenting may be necessary [12,14,23]. Hayek et al. showed that around 42% of patients developed TIPS dysfunction, including 15% who had more than one episode of TIPS dysfunction due to technical and clinical factors [24]. The technique of TIPS placement can be challenging in conditions of severe thrombosed portal or hepatic veins. Thus, the use of covered stents has been found to diminish the rate of TIPS dysfunction, maintain long-term shunt patency, and result in better clinical outcomes compared to bare metal stents [24]. The mechanism behind PTFE-covered stents involves inhibiting the growth of the inner layer of blood vessels, thereby maintaining stent patency [23]. If a TIPS occlusion is detected, a revision of the TIPS procedure is carried out. Rössle et al. reported that covered stent patency was achieved, with 1- and 5-year success rates of 93% and 74%, respectively [20]. Xingshun et al. reported that out of 22 patients who required TIPS revision, 6 patients had shunt dysfunction, and 2 patients died due to massive variceal bleeding following TIPS revision [18]. Han et al. showed that 17 patients required TIPS revision with stent placement; out of these patients, three required TIPS revision twice and one required it four times [25]. Tripathi et al. concluded that good clinical outcomes following the use of covered stents favour the long-term patency of TIPS, with less need for shunt re-interventions in BCS patients [15]. In our study, a covered stent was utilized for TIPS, and out of all patients, 47 (52.8%) required TIPS revision. Of these, only six patients (12.7%) needed more than 10 TIPS revisions. During the followup duration of 76 months, no patient died post TIPS procedure. Cumulative 1-, 3-, and 5-year TIPS stent patency rates standing at 98.8%, 97.9%, and 97.7% respectively, suggesting that TIPS can be an effective long-term treatment option for BCS. Within this cohort, there were no fatalities reported, in contrast to the study by Seijo et al. [26].

A notable limitation of our study is the absence of a controlled trial comparing TIPS with LTx. The favourable long-term survival rates post TIPS affirm its value as a primary treatment for BCS. The importance of TIPS in managing BCS is becoming more widely acknowledged, and LTx is now considered only after TIPS failure. Our experience aligns with the recognised benefits of TIPS for BCS patients.

## 6. Conclusions

Our study highlights that TIPS remains a highly effective and technically feasible treatment option for patients with BCS, demonstrating high rates of procedural success and favourable long-term outcomes. Despite the significant challenges associated with BCS, including the need for multiple TIPS revisions due to shunt dysfunction, the use of covered stents has markedly improved patency rates and reduced the frequency of re-interventions. Our findings corroborate the results of previous studies while also emphasizing the importance of individualized patient management and long-term followup to optimize clinical outcomes. Given the rarity of BCS and the complexity of its treatment, further large-scale, multi-centre studies are warranted to refine therapeutic strategies and enhance patient care in this challenging population.

## Figures and Tables

**Table 1:** Baseline characteristics of TIPS patients (N = 70).

Variables		Frequency (Percentage)
Age (years)	(mean ± SD)	37.7 ± 11.2
Age < 40 years		36 (51.4%)
Gender	Male	25 (35.7%)
	Female	45 (64.3%)
BMI		26.8 ± 6.8
MELD	(median)	18
Causes of BCS	MPD (JAK2 + ve)	23 (32.9%)
	Antiphospholipid syndrome	12 (17.1%)
	Vasculitis	2 (2.9%)
	Anti-thrombotic factor deficiency	7 (10%)
	Unknown	26 (37.1%)
Liver cirrhosis at diagnosis		14 (20%)
Comorbidities	Diabetes mellitus	6 (8.58%)
	Hypertension	3 (4.29%)
	Previous liver diseases	3 (4.29%)
	Heart disease	7 (10%)
	Kidney disease	5 (7.15%)
Associated IVC thrombosis		20 (28.6%)
PVT		15 (21.45%)

BCS: Budd–Chiari Syndrome; MPD: myeloproliferative disorder; BMI: Body Mass Index; MELD: Model for End-Stage Liver Disease; PVT: portal vein thrombosis; IVC: inferior vena cava.

**Table 2:** Clinical presentation of BCS patients who underwent TIPS (N = 70).

Clinical Presentation	Frequency (Percentage)
Abdominal pain	62 (88.6%)
Jaundice	62 (88.6%)
Decreased LOC	4 (5.72%)
Gastrointestinal bleeding	16 (22.8%)
Ascites	50 (71.5%)
ALF	2 (2.86%)
AKI	5 (7.15%)
Pulmonary symptoms	8 (11.44%)

LOC: level of consciousness; ALF: acute liver failure; AKI: acute kidney injury.

## Data Availability

The dataset used and/or analysed during the current study are available from the corresponding author upon reasonable request.
